# Circulating cell-free DNA fragmentation is a stepwise and conserved process linked to apoptosis

**DOI:** 10.1186/s12915-023-01752-6

**Published:** 2023-11-13

**Authors:** Dandan Zhu, Haihong Wang, Wei Wu, Shuaipeng Geng, Guolin Zhong, Yunfei Li, Han Guo, Guanghui Long, Qingqi Ren, Yi Luan, Chaohui Duan, Bing Wei, Jie Ma, Shiyong Li, Jun Zhou, Mao Mao

**Affiliations:** 1 Clinical Laboratories, Shenyou Bio, Zhengzhou, 450000 China; 2 Shanghai Institute of Hematology, CNRS-LIA Hematology and Cancer, Sino-French Research Center for Life Sciences and Genomics, State Key Laboratory of Medical Genomics, Rui Jin Hospital Affiliated to Shanghai Jiao Tong University School of Medicine, Shanghai, 200025 China; 3 Research & Development, SeekIn Inc, Shenzhen, 518000 China; 4 Department of Hepatobiliary and Pancreatic Surgery, Peking University Shenzhen Hospital, Shenzhen, 518000 China; 5 Clinical Laboratories, Sun Yat-Sen Memorial Hospital, Sun Yat-Sen University, Guangzhou, 510000 China; 6 School of Life Science and Technology, ShanghaiTech University, Shanghai, 201210 China; 7 Department of Molecular Pathology, The Affiliated Cancer Hospital of Zhengzhou University and Henan Cancer Hospital, Zhengzhou, 450003 China; 8 Yonsei Song-Dang Institute for Cancer Research, Yonsei University, Seoul, 03722 South Korea

**Keywords:** Circulating cell-free DNA, Shallow whole-genome sequencing, Size profiling of cfDNA, End preference

## Abstract

**Background:**

Circulating cell-free DNA (cfDNA) is a pool of short DNA fragments mainly released from apoptotic hematopoietic cells. Nevertheless, the precise physiological process governing the DNA fragmentation and molecular profile of cfDNA remains obscure. To dissect the DNA fragmentation process, we use a human leukemia cell line HL60 undergoing apoptosis to analyze the size distribution of DNA fragments by shallow whole-genome sequencing (sWGS). Meanwhile, we also scrutinize the size profile of plasma cfDNA in 901 healthy human subjects and 38 dogs, as well as 438 patients with six common cancer types by sWGS.

**Results:**

Distinct size distribution profiles were observed in the HL60 cell pellet and supernatant, suggesting fragmentation is a stepwise process. Meanwhile, C-end preference was seen in both intracellular and extracellular cfDNA fragments. Moreover, the cfDNA profiles are characteristic and conserved across mammals. Compared with healthy subjects, distinct cfDNA profiles with a higher proportion of short fragments and lower C-end preference were found in cancer patients.

**Conclusions:**

Our study provides new insight into fragmentomics of circulating cfDNA processing, which will be useful for early diagnosis of cancer and surveillance during cancer progression.

**Supplementary Information:**

The online version contains supplementary material available at 10.1186/s12915-023-01752-6.

## Background

Cell-free DNA (cfDNA) was initially discovered in human blood in the 1940s [1], and now, it has been detected in all body fluids [2]. Circulating cfDNA is known as fragments released mainly from apoptosis of normal hematopoietic cells. While the profile of circulating cfDNA in healthy individuals shows a modal size of 166 bp which bears correspondence to its association with nucleosome and linker histone H1, and a series of minor peaks exhibiting a 10-bp periodicity below 166 bp, tumor-derived DNA (circulating tumor DNA, ctDNA) tends to display a shorter modal size [3, 4]. In the last 20 years, cfDNA has been regarded as a novel source of various biological and pathological information such as pregnancy and cancer [5]. Although circulating cfDNA is now generally used as a non-invasive biomarker, the precise physiological process governing the DNA fragmentation and molecular profile of cfDNA remains obscure.

The works from Han et al. demonstrate that major nucleases including DNA fragmentation factor B (DFFB), DNASE1, and DNASE1L3 play important roles in the size profiling and base-end preference of circulating cfDNA fragmentation in mouse models [6–8]. In addition, it has been widely accepted that the fragmentation of cfDNA occurs in a non-random manner, which is associated with the positioning of nucleosomes and chromatin accessibility [9–13]. The fragmentation patterns observed in an individual’s cfDNA might contain evidence of the epigenetic landscapes of the cells giving rise to these fragments [9–13].

Plasma cfDNA in healthy individuals is a pool of highly degraded DNA fragments (a predominant peak at 166 bp and a series of subpeaks with a 10-bp periodicity below 166 bp) primarily derived from apoptosis of hematopoietic cells [11, 14]. In the current study, we took advantage of a human hematopoietic cell line HL60 to demonstrate the tight link between apoptosis and cfDNA fragmentation. The results indicated that the process of cfDNA fragmentation took place successively inside and outside apoptotic HL60 cells which gave rise to four predominant peaks corresponding to the nucleosomal units, as well as a series of 10-bp periodic sub-nucleosomal fragments below 167 bp. In addition, C-end preference of cfDNA fragments was observed in both apoptotic HL60 cell pellet and supernatant. Moreover, we scrutinized the cfDNA fragmentation profiles in a large cohort of healthy human subjects and cancer patients and found prominent differences between them. Our findings also suggest fragmentation process is conserved across mammals. Overall, our findings provide new insights into fragmentomics of cfDNA processing and profiling, which will be useful for early diagnosis of cancer and surveillance during cancer progression.

## Results

### DNA fragmentation of apoptotic cells is a two-step process which gives rise to major and minor peaks at different places

**Fig. 1:**
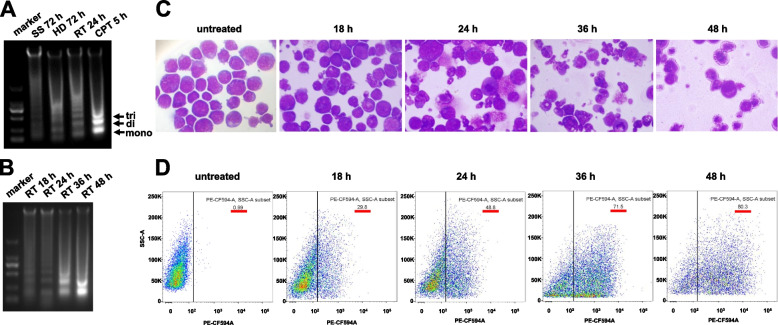
DNA fragmentation is tightly linked with cell apoptosis. **A** Electrophoresis analysis of the typical apoptotic DNA ladder extracted from HL60 cells cultured in different conditions including serum starvation (SS) for 72 h, high density (HD) for 72 h, room temperature (RT) for 14 h, and camptothecin (CPT) treatment for 5 h. Marker: 100 bp, 250 bp, 500 bp, 750 bp, 1000 bp, 2000 bp. 1.0% agarose gel. **B** Electrophoresis analysis of the apoptotic DNA ladder extracted from HL60 cells cultured at RT for 18 h, 24 h, 36 h, and 48 h. **C**, **D** May-Grunwald Giemsa (MGG) cell morphology and TdT-mediated dUTP nick-end labeling (TUNEL) cell apoptosis analyses of control and HL60 cells cultured at RT for 18 h, 24 h, 36 h, and 48 h

DNA ladder formation is the most well-known characteristic of apoptosis. Since the 1990s, the HL60 cell line has been regarded as an ideal model for studying cell apoptosis [15]. We therefore took advantage of the HL60 line, which served as a model system linking DNA fragmentation formed inside apoptotic cells (i.e., DNA ladder) with cfDNA fragments in plasma, to dissect the biological process of cfDNA fragmentation. HL60 cells were induced to apoptosis with either different conditions including exposure to serum starvation (SS), high density (HD), room temperature (RT) [16], or with apoptotic inducer such as camptothecin (CPT) [17]. Similar apoptotic DNA ladders were found for each treatment (Fig. 1A), confirming chromatin fragmentation is a characteristic of apoptosis as the ladders were composed of one, two, three, four, and multiple nucleosomal units.

Since the experiments above showed that the exposure to RT gave rise to a significant apoptotic DNA ladder, we collected the HL60 cells cultured at RT at different time points. As cells underwent apoptosis, an increasingly prominent DNA fragmentation ladder was observed (Fig. 1B). To further demonstrate the effects on apoptosis, May-Grunwald-Giemsa (MGG) staining and TdT-mediated dUTP nick-end labeling (TUNEL) were performed. The time course analyses showed that an increasing proportion of cells became TUNEL-positive and exhibited typical apoptotic features such as intact cell membrane, pyknosis, and nuclear fragmentation (Fig. 1C, D).

**Fig. 2:**
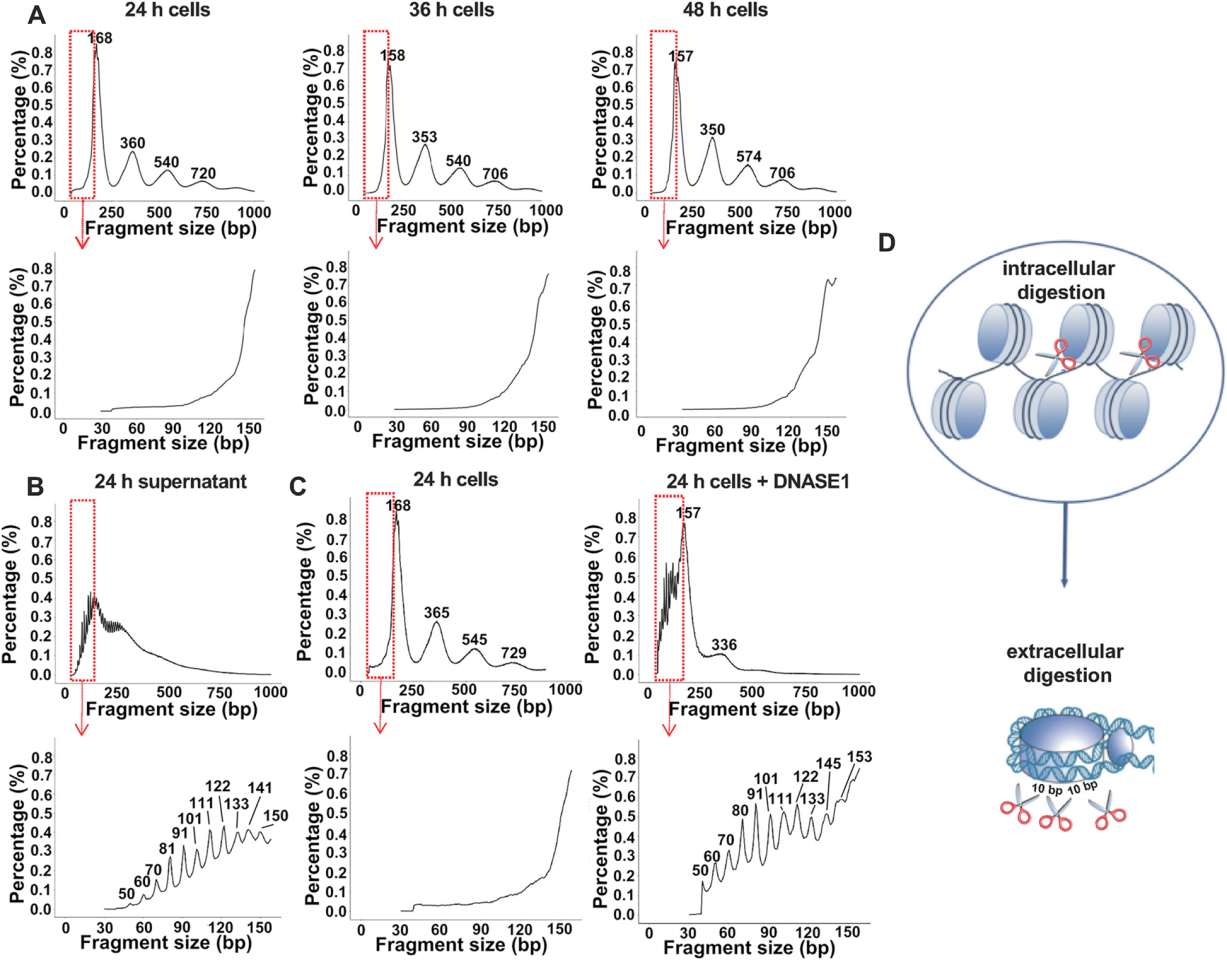
cfDNA fragmentation is a two-step process involving successive intracellular and extracellular digestion. **A** DNA size distribution profiles revealed by sWGS in pellets from HL60 cells exposed to RT for 24 h, 36 h, and 48 h. Bottom panels are enlarged views of the boxed areas in the top panels. **B** DNA size distribution profile in the supernatant of HL60 cells, which were exposed to RT for 24 h. **C** DNA size distribution profiles in the lysate of HL60 cells, which were exposed to RT for 24 h to induce apoptosis in advance, then treated with DNASE1. **D** Model of the stepwise cfDNA fragmentation process

Based on the observations above, shallow whole-genome sequencing (sWGS, 1 × coverage) was carried out to reveal the fragment size distribution of apoptotic HL60 cells with high resolution. The results indicated that the size distribution spread around the predominant peaks at ~ 167 bp, ~ 360 bp, ~ 540 bp, and ~ 720 bp (Fig. 2A), which was corresponding to the nucleosomal units (mono, di, tri, tetra). Nevertheless, the 10-bp periodic sub-nucleosomal fragments below 167 bp which are observed in plasma could not be found (Fig. 2A). Such phenomena imply that the linker region between core nucleosomes, rather than the DNA wrapping around the nucleosome core, is cut by the nucleases inside the apoptotic cells.

Next, to unravel the origin of the ~10-bp periodicity, we examined the size distribution of DNA fragments in the supernatant of HL60 cells. As expected, sWGS analysis displayed a series of peaks with a clear 10-bp periodicity (Fig. 2B), suggesting that the nucleosomes released from the apoptotic HL60 cells could be further digested with DNases. Since the serum added in the culture medium had been heat-inactivated, the active DNases in the supernatant should be produced by HL60 cells and secreted into the extracellular milieu.

To further illustrate that the 10-bp periodicity was generated from nucleosomes, the pellet of apoptotic HL60 cells (which were cultured at RT for 24 h to induce apoptosis in advance) was lysed to release the nucleosomes therein, then the lysate was subjected to a treatment with DNASE1. As anticipated, sWGS analysis revealed a similar 10-bp periodicity (Fig. 2C).

Finally, to replicate the findings from HL60 cells in other cell types, particularly non-cancerous cells, GM12878 and NIH3T3 cell lines were used. Unfortunately, although GM12878 cells were killed with diverse stimuli, a typical apoptotic DNA ladder could not be produced (Additional file 1: Fig. S1). By contrast, NIH3T3 cells were induced to apoptosis with H_2_O_2_ treatment (Additional file 2: Fig. S2A). As expected, similar DNA fragmentation profiles as observed inside and outside HL60 cells were revealed by sWGS analysis as well (Additional file 2: Fig. S2B).

Combining the data above, we could draw the conclusion that cfDNA fragmentation is a two-step process involving intracellular and extracellular digestions which eventually gives rise to major and minor cfDNA peaks (Fig. 2D).

### C-end preference is observed in cfDNA fragments in supernatant and within apoptotic HL60 cells

DFFB, DNASE1, and DNASE1L3 are three major endonucleases that contribute to the size profile of circulating cfDNA [18–20]. While DFFB is responsible for the oligo-nucleosomal fragmentation of chromatin inside apoptotic cells, DNASE1 and DNASE1L3 account for further digestion of the cfDNA fragments in circulation.

Typical C-end preference is found in circulating cfDNA in plasma. To clarify whether this typical cfDNA profile was generated ‘‘as is’’ from apoptotic cells or produced through further digestion in plasma, Han et al. investigated the newly released cfDNA from apoptotic leukocytes in the presence of the anticoagulant EDTA inhibiting the activity of plasma DNASE1 and DNASE1L3 [6]. After incubation of whole blood at RT for 6 h to induce cell death, they found the C-end predominance in typical cfDNA was greatly diminished while A-end preference was increased in the newly released cfDNA [6]. In combination with the results from several *DNASE*-deficient mice, they concluded that the newly released cfDNA fragments are A-end enriched, which are produced with DFFB (Prefers A > G >  > C&T), DNASE1L3, and other intracellular enzymes. DNASE1L3 (Prefers C > T >  > A&G) further digests these fragments in plasma, and gives rise to the typical profile with C-end predominance in cfDNA [6].

**Fig. 3:**
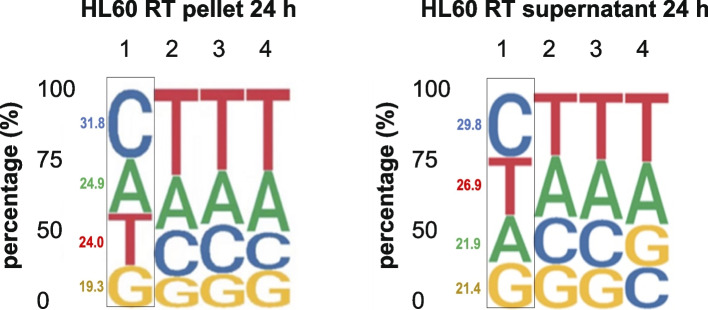
Base content proportions at the 5′ end of cfDNA fragments in RT induced apoptotic HL60 cell pellet and supernatant. **A** Human genome is composed of 29.5% A, 29.6% T, 20.4% C, and 20.5% G. The proportion of fragments with A-, T-, C-, and G-end in HL60 cell pellet is 24.9%, 24.0%, 31.8%, and 19.3%. The proportion of C-end fragments is significantly increased. The fractions of the first allele were indicated. **B** C-end preference is also observed in HL60 supernatant. The proportion of fragments with A-, T-, C-, and G-end is 21.9%, 26.9%, 29.8%, and 21.4%. The fractions of the 1st allele were indicated

Although inactivation of DNASE1L3 caused a significant reduction of C-end preference in the newly released cfDNA, the proportion of C-end fragments remained predominant [6]. It is worth noting that the expression of *DNASE1L3* is absent in HL60 cells (Human Protein Atlas database, and Additional file 3: Fig. S3), and C-end predominance of the 5′ end of DNA fragments was found in both HL60 cell pellet and supernatant (Fig. 3A, B).

Collectively, these findings indicate that even in the absence of DNASE1L3, C-end preference exists inside and outside apoptotic HL60 cells.

### Plasma cfDNA profile is characteristic and conserved across mammals

**Fig. 4:**
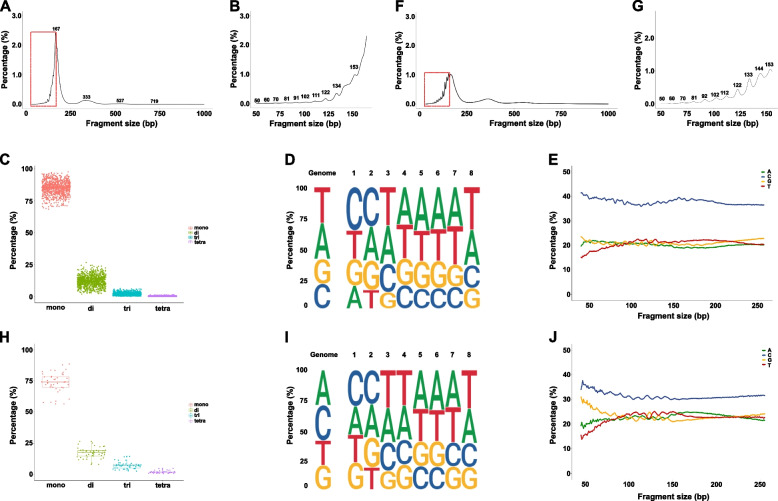
Characteristics of plasma cfDNA fragmentation in 901 healthy human and 38 dog individuals. **A** The size distribution of cfDNA was inferred by paired-end sequencing of plasma samples from 901 healthy human subjects. The predominant peaks at 167 bp, and three additional peaks at 333 bp, 527 bp, and 719 bp were marked. **B** The enlarged view of the boxed areas in panel **A**, indicating the ten most distinguishable periodic subpeaks around 50, 60, 70, 81, 91, 102, 111, 122, 134, and 153 bp. **C** Proportions of the fragments representing mono-, di-, tri-, and tetra-nucleosomes are 85.0%, 12.1%, 2.3%, and 0.4%, respectively. **D** Human genome is composed of 29.5% A, 29.6% T, 20.4% C, and 20.5% G (reference). The proportion of fragments with 5′ A-, T-, C-, and G-ends in circulating cfDNA is 18.8%, 23.3%, 35.9%, and 22.0% in average in 901 healthy human individuals (Position 1). **E** C-end preference is found in all fragment sizes (50–250 bp) in 901 healthy human individuals. **F** The size distribution of cfDNA was inferred by paired-end sequencing of plasma samples from 38 healthy dogs. The predominant peaks at 163 bp, and three additional peaks at 362 bp, 547 bp, and 728 bp were marked. **G** The enlarged view of the boxed areas in panel **F**, indicating the ten most distinguishable periodic subpeaks around 50, 60, 70, 81, 92, 102, 112, 122, 133, 144, and 153 bp. **H** Proportions of the fragments representing mono-, di-, tri-, and tetra-nucleosomes are 74.2%, 17.2%, 6.9%, and 1.7%, respectively. **I** Dog genome is composed of 29.5% A, 20.5% T, 29.5% C, and 20.5% G (reference). The proportion of fragments with 5′ A-, T-, C-, and G-ends in circulating cfDNA is 23.7%, 23.3%, 30.3%, and 22.7% in average in 38 healthy dogs (Position 1). **J** C-end preference is in all fragment sizes (50–250 bp) in 38 healthy dogs

The sWGS analysis enabled us to interrogate the size distributions of cfDNA in the human individuals as well. We examined the plasma samples from 901 healthy human individuals and found that cfDNA fragments displayed a distribution with a predominant peak size of 167 bp (85.0%) (Fig. 4A, C). Meanwhile, we also observed three additional peaks at 333 bp (12.1%), 527 bp (2.3%), and 719 bp (0.4%), respectively (Fig. 4A, C). The four peaks are representing mono-, di-, tri-, and tetra-nucleosomes corresponding to what we observed from the apoptotic cells above.

In addition to the four major peaks, distinct fragmentation features including intensified amplitudes of approximately 10-bp oscillations in the 50 to 153 bp size range with periodic peaks and troughs were simultaneously observed (Fig. 4B). A quantitative proxy was established to demonstrate that the amplitudes of the ten most distinguishable periodic subpeaks were ~ 50, 60, 70, 81, 91, 102, 111, 122, 134, and 153 bp, which was nearly identical to the positions of the minor peaks found in HL60 supernatant (~ 50, 60, 70, 81, 91, 101, 111, 122, 133, 141, and 150 bp), except for ~ 144–146 bp was a plateau (Fig. 4B).

Moreover, we analyzed the base content proportions at the 5′ end motifs of plasma cfDNA fragments. An overall C predominance was found at the first and the second base of the 5′ ends of the cfDNA fragments (Fig. 4D), which is in line with the findings in mice [5], and the preferred C-end was found across all the fragments in the first major peak (50–250 bp) (Fig. 4E).

To further demonstrate that the cfDNA fragmentation process is conserved across mammals, 38 samples from the plasma of healthy dogs were analyzed. cfDNA distribution profile showed that the minor peaks were ~ 50, 60, 70, 81, 92, 102, 112, 122, 133, 144, and 153 bp, which was in line with the 10-bp sub-nucleosomal periodicity observed in humans (Fig. 4F, G). Yet, the dominant 167 bp peak was replaced by an additional peak at 163 bp (Fig. 4F, G), which would be caused by a stronger activity of DNases in canine plasma. Meanwhile, three additional major peaks at 362 bp (17.2%), 547 bp (6.9%), and 728 bp (1.7%) were observed (Fig. 4F, H). Besides, it is worth noting that although the base composition in the dog genome is different with that in humans, a similar C predominance at the first and the second base of the 5′ ends of the cfDNA fragments was also revealed (Fig. 4I, J).

Combining our observations with the fact that a similar cfDNA fragmentation profile with a major peak and a series of minor peaks in murine plasma [6], it seems that the circulating cfDNA profile is characteristic and conserved across mammals.

### The characteristics of cfDNA fragmentation in cancer patients are distinct with healthy individuals

**Fig. 5:**
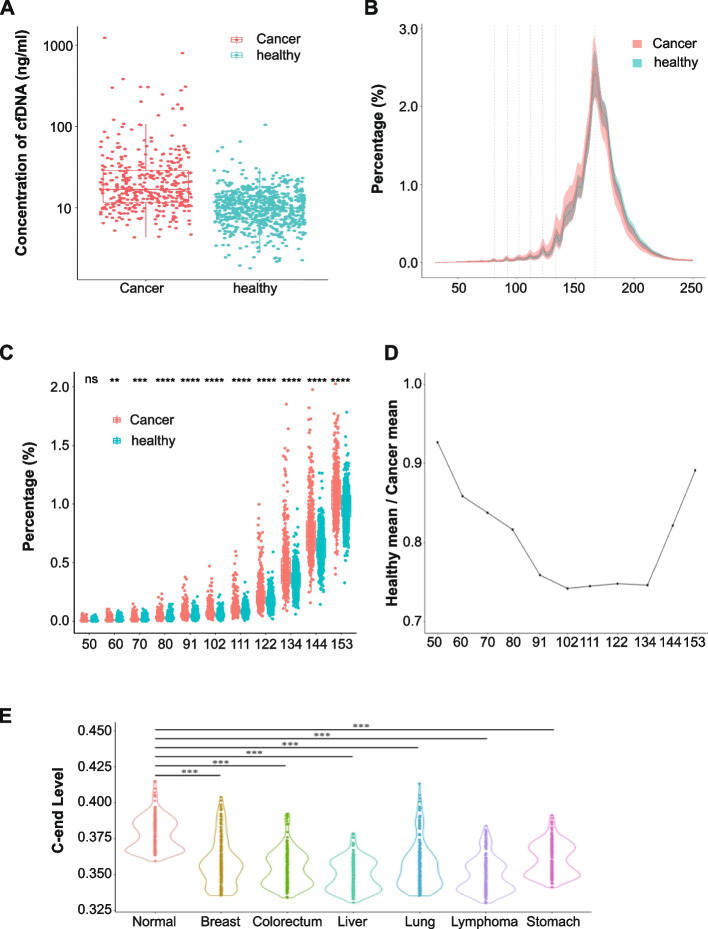
Distinct characteristics in cancer patients. **A** Concentration of cfDNA in plasma in 438 cancer patients and 901 healthy individuals. **B** The size distribution of cfDNA was inferred by paired-end sequencing of plasma samples from cancer patients and healthy individuals. **C** Scatter plot of the ratio of P50, P60, P70, P81, P91, P102, P111, P122, P134, P144, and P153 from cancer patients and healthy individuals. ns: not significant, **P < 0.01, ***P < 0.001, ****P < 0.0001 (*U*-test). **D** Line plots show the ratio of reads falling into each peak ± 1 bp ranges of eleven periodic peaks (50, 60, 70, 81, 91, 102, 111, 122, 134, 144, and 153 bp) from cancer patients and healthy individuals. **E** Violin plots of the percentage of the cfDNA fragments of different sizes with C-end in cancer patients and healthy subjects. Statistical significance of the decreased C-end preference in cancer patients was evaluated by paired *t*-test (***p < 0.001)

Circulating tumor DNA (ctDNA) is now generally used as a non-invasive biomarker to detect cancer [21–26]. To display the ctDNA profile in cancer patients, we collected plasma samples from 438 patients with six different types of cancer (breast 67, colorectum 54, liver 128, lung 39, lymphoma 98, stomach 52). Compared with the healthy human individuals, a generally higher content of plasma cfDNA was detected (Fig. 5A). Meanwhile, distinct cfDNA profiles were revealed by sWGS analysis. To better display the distinction of cfDNA profiles in cancer and normal plasmas (Fig. 5B), we carried out a set of in-depth analyses. A quantitative proxy was established to estimate the amplitudes of the eleven most distinguishable periodic peaks (~ 50, 60, 70, 81, 91, 102, 111, 122, 134, 144, and 153 bp) individually: the percentage of sequencing reads falling into the peaking size ± one bp range against total fragment reads from the samples. As shown in Fig. 5C and D, except for the peak at ~ 50 bp, the percentage of reads from all of the other peaks was significantly higher in the cancer group compared with the healthy group.

Jiang et al. found that the abundance of cfDNA fragments with CCCA motif (the most frequent motif in plasma cfDNA of healthy human individuals) was much lower in hepatocellular carcinoma (HCC) patients than in the subjects without HCC [21]. This kind of aberrant end motifs was also observed in patients with other types of cancer such as lung cancer, colorectal cancer, nasopharyngeal carcinoma, etc. [22, 27]. In concert with these results, we found the cfDNA fragments with C-end were also significantly decreased in patients with the six cancer types (Fig. 5E).

Based on these observations, one can conclude the cfDNA profiles in the healthy individuals and the patients with six types of cancer are quite different. Overall, our findings provide new insights into fragmentomics of cfDNA processing and profiling, which will be useful for early diagnosis of cancer and surveillance during cancer progression.

## Discussion

From the current work on cfDNA profiles in human leukemic cell line HL60 and mouse nontumoral fibroblast cell line NIH3T3, we can piece out a model that outlines the cfDNA fragmentation process. cfDNA fragmentation is a stepwise process which takes place successively inside and outside the apoptotic cells. The peaks representing the nucleosomal units observed in the apoptotic cells suggest the cleavage is inter-nucleosomal. The 10-bp periodicity found in the supernatant suggests the digestion is intra-nucleosomal, which should be accomplished by the DNases secreted from cells as the serum added in the culture medium has been heat-inactivated.

In a previous study, the bi-potential HL-60/S4 cell line was induced to differentiate toward neutrophil or macrophage lineage upon treatment with RA or TPA [28]. The authors performed ChIP-seq analyses by histone H3 and its modifications and found a genome-wide change of nucleosome occupancies as HL-60/S4 cells differentiate. Based on these observations, a conclusion was drawn that chromatin changes during HL-60/S4 differentiation appeared to be more localized to regulatory regions such as promoters and enhancers. Although cfDNA sequences may reflect chromatin features at the gene-regulation level, the results obtained from this study are not comparable to those from our work due to different experimental strategies (ChIP-seq with H3 vs sWGS).

The observations from Han et al. reveal that plasma cfDNA is generated first with intra-cellular DFFB and DNASE1L3 during apoptosis, then cfDNA molecules that have escaped from phagocytosis are subsequently digested by circulating DNASE1L3 and DNASE1 in mouse models [6]. The 10-bp periodicity in the short circulating cfDNA fragments can be seen among all types of nuclease knockout mice (*Dffb*^*−/−*^, *Dnase1l3*^*−/−*^, and *Dnase1*^*−/−*^), implying a single particular endonuclease would not be responsible for the sub-nucleosomal cleavage.

In the Human Protein Atlas database (https://www.proteinatlas.org) it is shown that major endonucleases such as DFFB and DNASE1 are expressed in HL60 cells. Considering the fact that the 10-bp periodicity can only be found in supernatant, our findings suggest that although these endonucleases exist within HL60 cells, they do not play a role in DNA fragmentation at the intra-nucleosomal level. Perhaps the inter-nuclesomal cleavage to rapidly digest a large of amount of chromatin, rather than degrade the DNA fragments into smaller ones to prevent autoimmunity, is the priority during apoptosis.

Han et al. also demonstrated that each nuclease has its own preference for the end of fragment, and C-end preponderance is revealed in typical circulating cfDNA in mice [6]. Consistently, our results show that C-end is predominant in both healthy human and dog individuals. Moreover, such preference is also observed in HL60 cells with different treatments such as CPT and serum starvation (data not shown).

Overall, we analyze the DNA fragmentation profile of a large cohort of healthy human donors in the present work. To our knowledge, the sample size is largest in the existing literature [21–27, 29–33]. In addition, we also analyzed plasma cfDNA in 38 healthy dogs. Note that although the composition of the genome is quite distinct in humans, mice [6], and dogs, their cfDNA profiles and end preferences are nearly identical. Hence, the cfDNA fragmentation process would be conserved across mammals since the cleavage is based on nucleosome structure, which is highly conserved in the eukaryote landscape.

Furthermore, the cfDNA profile in the patients with six types of cancer displays a significant increase of fragments less than 167 bp, and a reduction with C-end preference, which is in line with our previous observations in HCC [4] and the work from the others [21, 22, 29]. Statistical analyses further indicate that the subpeaks are significantly higher in the cancer group compared with the healthy group. Although the shortening of cfDNA fragments and lowered C-end predominance have been adopted as two characteristics to distinguish cancer patients and normal individuals, the reason behind remains enigmatic.

In *Dnase1l3* knockout mice, Serpas et al. demonstrated that the deficiency of *Dnase1l3* can lead to an increase of short DNA molecules below 120 bp as well as long fragments above 250 bp (which are shown as reminiscent of nucleosomal units), and a remarkable reduction in the most common 4-mer end motifs CCNN in plasma cfDNA (the top six motifs are CCCA, CCTG, CCAG, CCAA, CCAT, and CCTC) [8]. Besides, Jiang et al. found that the transcription level of *DNASE1L3* was dramatically decreased in tumor tissues compared with adjacent non-malignant liver tissues. Combining with the fact that *DNASE1L3* is generally downregulated across multiple cancers (The Cancer Genome Atlas, TCGA database) [34], inactivation of DNASE1L3 is proposed to be the reason underlying the aberrant cfDNA profile in cancer patients.

In a recent work of An et al., cfDNA molecules of different DNA methylation levels showed drastically different sizes and end distributions. Actually, distinct cfDNA profiles with a higher proportion of short fragments and lower C-end preference were revealed by sWGS analysis in our patients with various cancer types. Given the facts that DNA methylation is known to be abnormal in all forms of cancer and DNASE1L3 is dramatically decreased in tumor tissues, a DNA methylation-nuclease preference-cutting end-size distribution axis would serve as a key regulator of cfDNA fragmentation [13].

In addition, psychosocial and physical stress conditions can also induce cfDNA release in healthy individuals [35]. Nevertheless, the functional role of such cfDNA release remains obscure. Hence, more investigations are needed to explore the mechanism of cfDNA formation under different conditions such as health, stress, pregnancy, and diseases in the future.

## Conclusions

We took advantage of the HL60 cell line as a model system to study cfDNA fragmentation. Based on this model a two-step hypothesis was proposed by distinct cfDNA fragmentation patterns inside and outside HL60 cells. We also compared cfDNA profiles in a large cohort of healthy human/dog individuals, and cancer patients, and observed more minor peaks and less fragments with C-end in the latter group. Our findings can be used as a reference for clinical applications.

## Methods

### Cell culture, treatment, and preparation of cfDNA samples

Human promyelocytic leukemia HL60 cells were grown at 37 °C in the presence of 5% CO_2_ in RPMI 1640 medium supplemented with 10% fetal bovine serum (GIBCO, Life Technologies, Australia). The cell density was 2–4 × l0^5^/ml. To expose cells to room temperature (RT), 5 ml of HL60 cells were placed at RT, and recovered for analysis at time indicated. To expose cells to high density, l0^6^ HL60 cells were cultured in 1 ml medium. CPT (Sigma, Shanghai, China) was dissolved in DMSO and added in the medium, the final concentration was 10 µM. Mouse NIH3T3 fibroblasts were grown at 37 °C in the presence of 5% CO_2_ in DMEM medium supplemented with 10% fetal bovine serum (GIBCO, Life Technologies, Australia), and induced to apoptosis with 1000 µM H_2_O_2_ for 96 h.

Apoptotic DNA ladder from each sample was extracted with Apoptotic DNA Ladder Extraction Kit (Beyotime, Shanghai, China). Ten microliters of DNA was loaded on 1% agarose gel and visualized under UV light. The rest of the DNA was subjected to construct sequencing library. DNA in supernatant was extracted by using a QIAamp Circulating Nucleic Acid kit (QIAGEN, Hilden, Germany) following the manufacturer’s instructions, and subjected to library construction.

For the lysate, 5 ml of 2–4 × l0^5^/ml HL60 cells were cultured at RT for 24 h to induce apoptosis, then cells were centrifuged and resuspended in 500 µl PBS. After a vigorous vortex, the tubes were centrifuged at the maximum speed for 5 min and the cell debris was removed. The lysate was treated with 1 U DNASE1 (Takara, Shanghai, China) for 30 min at 37 °C. DNA was then extracted from the lysate using a QIAamp Circulating Nucleic Acid kit (QIAGEN, Hilden, Germany) following the manufacturer’s instructions, and subjected to construct sequencing library.

### Detection of HL60 cells undergoing apoptosis

Cytospins of wild type and apoptotic HL60 cells were prepared by cytocentrifugation at 1000 rpm in a Shandon Cytospin 3 centrifuge for 5 min. The slides were then methanol fixed, stained with May-Grünwald stain for 15 min followed by 30 min in 5% Giemsa stain (all from Merck, Darmstadt, Germany). Slides were then read under a microscope.

Apoptosis of HL60 cells was measured using a terminal-deoxynucleo-tidyltransferase (TdT)-mediated deoxyuridine triphosphate (dUTP) nick end labeling (TUNEL) technique. The “In situ Cell Death Detection Kit, AP” (Roche), which contains calf thymus TdT, fluorescein-dUTP and an alkaline phosphatase anti-fluorescein sheep Fab fragment, was used according to the manufacturer’s instruction. The samples were then subjected to FACS analysis to evaluate cell apoptosis. PE-CF594 was added to the cells (5 μl/tube) and incubated at 37 °C for 2 h. The number of apoptotic cells was counted by flow cytometry.

### Library construction and sequencing of HL60 and NIH3T3 cell lines

The integrity and concentration of DNA were determined by Qubit 4.0 fluorometer dsDNA HS Assay (Thermo Fisher Scientific, Waltham, USA). About 0.5–1 μg high-quality DNA sample was used to construct sequencing library. Sequencing libraries were prepared with KAPA Hyper Prep Kit (Kapa Biosystems, Wilmington, USA) according to the manufacturer’s protocol. The cohesive ends were repaired into blunt ends. The 3’ ends of the fragments were additionally adenylated with a single A, allowing hybridizing ligation to the 3’ overhanging T of sequencing adapters. After purification and ligation to the adapters, the resulting DNA fragments were selected using AMPure XP beads (Beckman Coulter, Krefeld, Germany) for a desired size of ~ 420 bp, followed by PCR amplification. Subsequently, purified library was sequenced on NovaSeq 6000 System (Illumina, San Diego, USA) for 2 × 150 bp paired-end sequencing for whole genome sequencing (WGS) at 1 × coverage.

### Blood sample collection and processing

Nine hundred one healthy human subjects and 438 newly diagnosed cancer patients were enrolled in the study. 8 ml peripheral blood from each individual was collected in Cell-Free DNA BCT® blood collection tubes (STRECK, La Vista, NE, USA). Blood samples were centrifuged at 1600 g for 10 min at 4 °C and the supernatants were collected, and then were further centrifuged at 16,000 g for 10 min at 4 °C. cfDNA was extracted from at least 2 ml plasma using QIAamp Circulating Nucleic Acid Kit (QIAGEN, Hilden, Germany) following the manufacturer’s instructions. cfDNA was subjected to library construction by KAPA Hyper Prep Kit according to the manufacturer’s protocol. Prepared libraries were sequenced on Illumina X Ten or NovaSeq system with 2 × 150 bp paired-end sequencing for WGS at ~ 3 × coverage.

Thirty-eight healthy dogs were enrolled in the study and 4 ml peripheral blood from each dog was collected in Boomingshing cfDNA Blood Collection Tubes (Shenzhen, China). The same protocols and instruments were used for plasma prep, cfDNA extraction, library prep, and sequencing as for human blood samples. The coverage of WGS was at ~ 1 × .

### Sequence alignment

Clean sequencing reads were mapped to human reference genome (UCSC hg38, https://hgdownload.soe.ucsc.edu/goldenPath/hg38/bigZips/), mouse reference genome (GRCm38.p4, https://ftp.ncbi.nlm.nih.gov/genomes/all/GCF/000/001/635/GCF_000001635.24_GRCm38.p4/GCF_000001635.24_GRCm38.p4_genomic.fna.gz), or dog reference genome (Cfamiliaris.UCSC.canFam3, https://hgdownload.soe.ucsc.edu/goldenPath/canFam3/bigZips/) respectively with BWA-MEM (v0.7.17) after cutting adapters (cutadapt v2.10), removing low quality reads and N reads, and trimming 9 bp from the read’s 5′ end. Sambamba (v0.6.8) was used to mark duplication and GATK (v4.0.12.0) was used for base quality score recalibration. Alignment quality metrics were computed using picard (v2.18; CollectWgsMetrics, CollectGcBiasMetrics, CollectMultipleMetrics). The BWA (version: 0.7.17) MEM mode was used for mapping the sequencing reads, with default parameters.

### Fragment size analysis

Read pairs with a MAPQ score below 30 for either read or PCR duplicates were removed. Subsequently, cfDNA fragment size was calculated based on the mapping position of the remaining paired-end reads. We summarized the number of cfDNA fragment within each fragment size and depicted the distribution of cfDNA fragment size.

### End motif analysis

The identification of cfDNA fragment end motifs was described by Jiang et al. [36]. In brief, after filtering out low-quality (MAPQ < 30) or duplicated reads, the end motifs were identified using the first 8-nucleotide sequence on each 5′ fragment end (Watson and Crick strands) of plasma DNA after alignment to human reference genome (UCSC hg38). The base content proportions of the end motifs were calculated at each position. The end motifs were grouped based on fragment size, then calculate the base content proportions of at the first position of end motifs under each fragment size.

### Statistics

All statistical analyses were performed using R statistical software (https://www.r-project.org, version 4.1.2). Delong’s test was used to compare the difference of AUC value.

### Supplementary Information

**Additional file 1: Fig. S1. **Electrophoresis of DNA extracted from GM12878 cells. Although GM12878 cells were killed with various stimuli including room temperature (RT, ~22℃, 48h), low temperature (4℃, 48h), heat shock (42℃, 1h) , serum starvation (SS, 72h), high density culture (HD, 72h), UV light (1h), camptothecin (CPT, 10µM, 5h), arsenic trioxide (ATO, 1µM, 72h), carbonyl cyanide m-chlorophenylhydrazine (CCCP, 10µM, 1h), puromycin (Puro,* 5*µ*g/ml*, 72h), and hygromycin (Hygro, 1000µg/ml, 72h), no typical DNA ladder could be found. Marker: 100 bp, 250 bp, 500 bp, 750 bp, 1000 bp, 2000 bp. 1.0 % agarose gel.

**Additional file 2: Fig. S2.** DNA ladder and cfDNA profiles from NIH3T3 cells. (A) Electrophoresis analysis of the apoptotic DNA ladder extracted from NIH3T3 cells treated with H_2_O_2_ (1000 µM) for 96 h. Marker: 100 bp, 250 bp, 500 bp, 750 bp, 1000 bp, 2000 bp. 1.0 % agarose gel. (B) DNA size distribution profiles revealed by sWGS in pellets and supernatant from NIH3T3 cells treated with H_2_O_2_.

**Additional file 3: Fig. S3.** RT-PCR analysis of *DNASE1L3 *expression. RT-PCR analysis indicated *DNASE1L3* was not expressed in HL60 cells. Marker: 100 bp, 250 bp, 500 bp, 750 bp, 1000 bp, 2000 bp. 1.0 % agarose gel. Lane 1-3: PCR products with three different pairs of primers on a *DNASE1L3 *plasmid. Lane 4-6: 40 rounds of PCR with the same primers failed to amplify *DNASE1L3 *in HL60 cells.

## Data Availability

All data generated or analyzed during this study are included in this published article, its supplementary information files, and publicly available repositories. The data from the HL60 cell line are now available at https://www.ncbi.nlm.nih.gov/bioproject/PRJNA963064 with an open access mandate. The data from NIH3T3 cell line are available at https://ngdc.cncb.ac.cn/bioproject/browse/PRJCA017828 with an open access mandate. Sequencing data of the healthy individuals and cancer patients, as well as clinical information about these patients are available with controlled access at https://ngdc.cncb.ac.cn/bioproject/browse/PRJCA017828.

## Abbreviations

cfDNA: Circulating cell-free DNA
sWGS: Shallow whole-genome sequencing
ctDNA: Circulating tumor DNA
SS: Serum starvation
HD: High density
RT: Room temperature
CPT: Camptothecin
MGG: May-Grunwald-Giemsa
TUNEL: TdT-mediated dUTP nick-end labeling
DFFB: DNA fragmentation factor B
