# Supplementary figures and images for: Circulating cell-free DNA fragmentation is a stepwise and conserved process linked to apoptosis
Source: BMC Biol. 2023 Nov 13;21:253. doi: 10.1186/s12915-023-01752-6 (PMC10642009; doi:10.1186/s12915-023-01752-6)

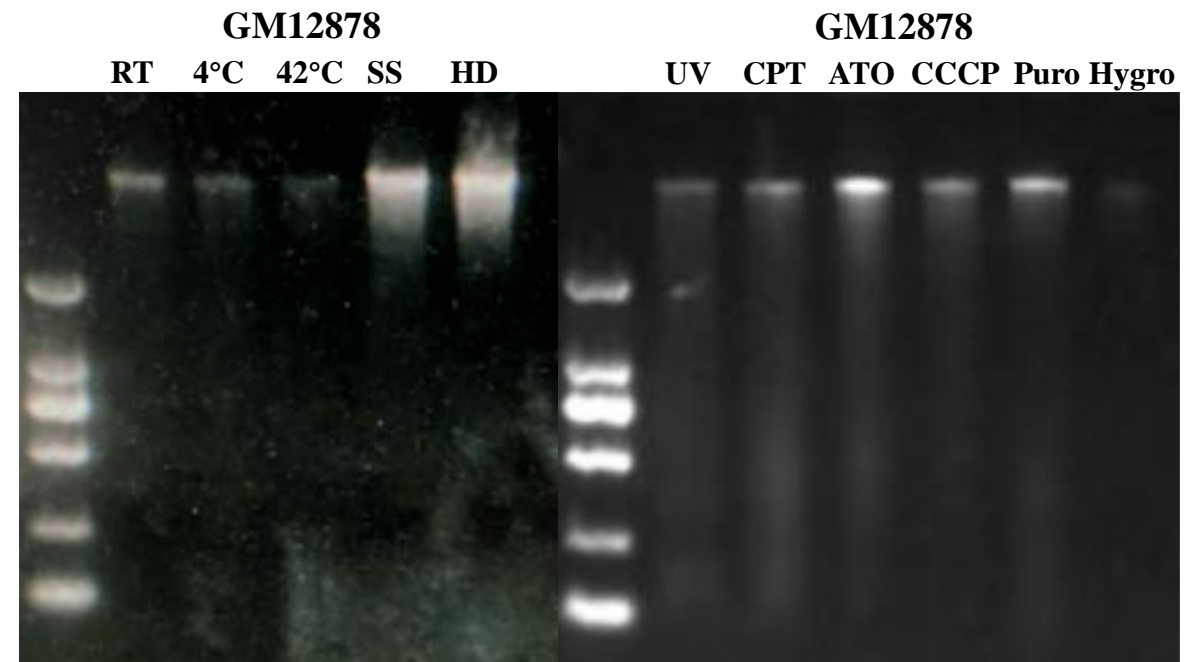

Supplement: Supplementary file 1 — Additional file 1: Fig. S1. Electrophoresis of DNA extracted from GM12878 cells. Although GM12878 cells were killed with various stimuli including room temperature (RT, ~22℃, 48h), low temperature (4℃, 48h), heat shock (42℃, 1h) , serum starvation (SS, 72h), high density culture (HD, 72h), UV light (1h), camptothecin (CPT, 10µM, 5h), arsenic trioxide (ATO, 1µM, 72h), carbonyl cyanide m-chlorophenylhydrazine (CCCP, 10µM, 1h), puromycin (Puro, 5µg/ml, 72h), and hygromycin (Hygro, 1000µg/ml, 72h), no typical DNA ladder could be found. Marker: 100 bp, 250 bp, 500 bp, 750 bp, 1000 bp, 2000 bp. 1.0 % agarose gel. [file 12915_2023_1752_MOESM1_ESM.pdf]

**A**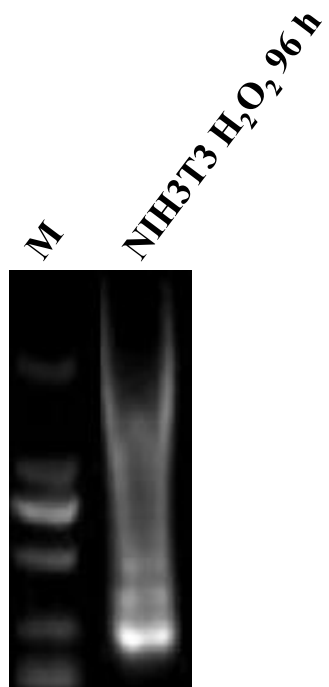**B**

NIH3T3 cells

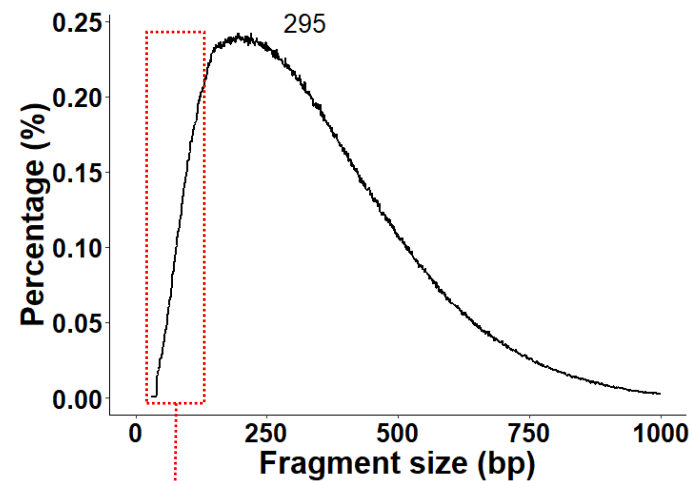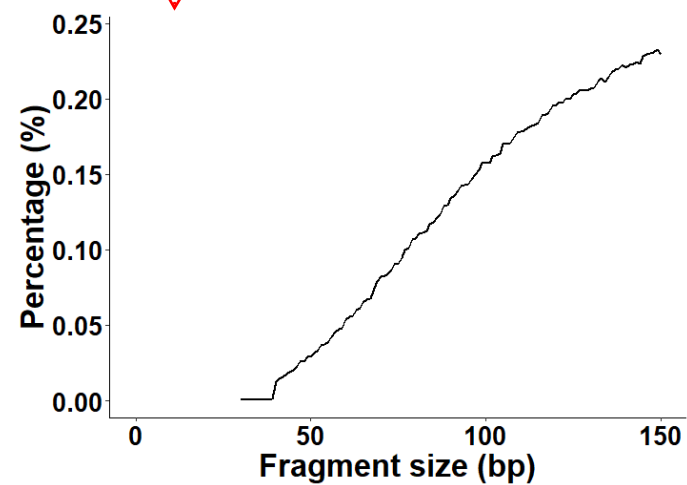

NIH3T3 supernatant

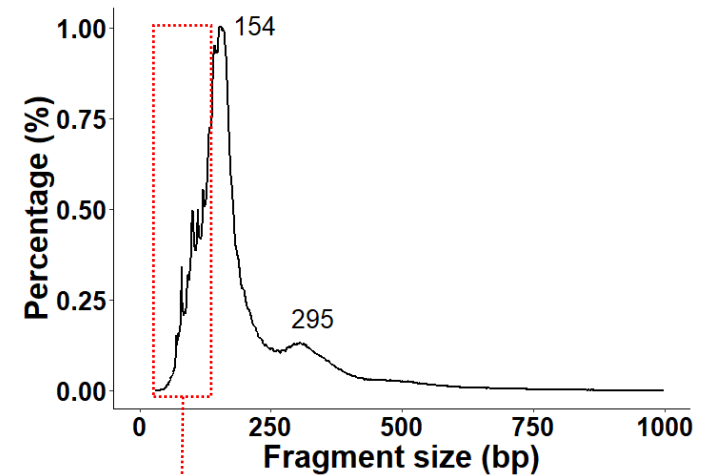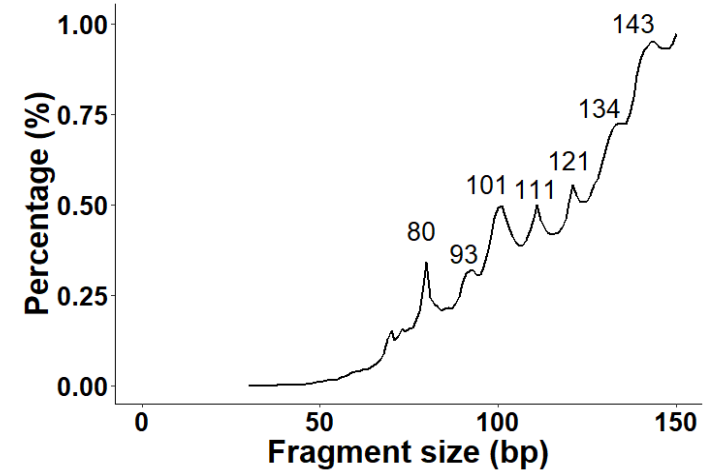

Supplement: Supplementary file 2 — Additional file 2: Fig. S2. DNA ladder and cfDNA profiles from NIH3T3 cells. (A) Electrophoresis analysis of the apoptotic DNA ladder extracted from NIH3T3 cells treated with H2O2 (1000 µM) for 96 h. Marker: 100 bp, 250 bp, 500 bp, 750 bp, 1000 bp, 2000 bp. 1.0 % agarose gel. (B) DNA size distribution profiles revealed by sWGS in pellets and supernatant from NIH3T3 cells treated with H2O2. [file 12915_2023_1752_MOESM2_ESM.pdf]

**M 1 2 3 4 5 6 M**

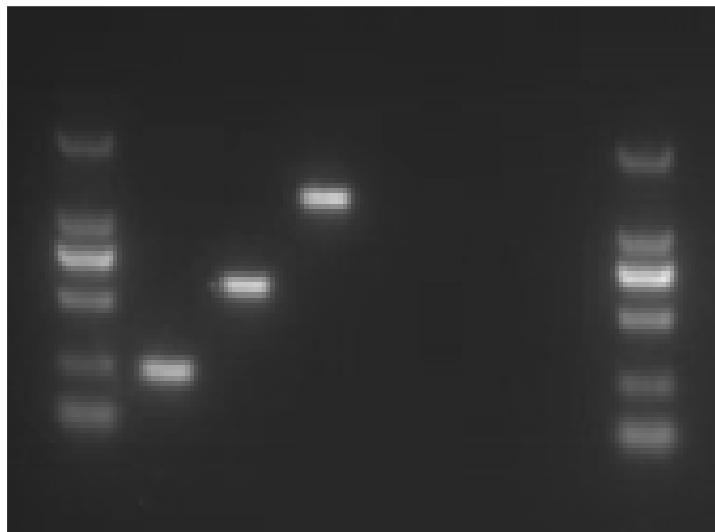

Supplement: Supplementary file 3 — Additional file 3: Fig. S3. RT-PCR analysis of DNASE1L3 expression. RT-PCR analysis indicated DNASE1L3 was not expressed in HL60 cells. Marker: 100 bp, 250 bp, 500 bp, 750 bp, 1000 bp, 2000 bp. 1.0 % agarose gel. Lane 1-3: PCR products with three different pairs of primers on a DNASE1L3 plasmid. Lane 4-6: 40 rounds of PCR with the same primers failed to amplify DNASE1L3 in HL60 cells. [file 12915_2023_1752_MOESM3_ESM.pdf]
